# Mortality for October

**Published:** 1866-11

**Authors:** 


### MORTALITY FOR OCTOBER.

*Causes of Death.*—Accident, 11; Bronchitis, 1; Cancer, 2; Childbed, 3; Cholera, 673; Cholera Morbus, 15; Cholera Infantum, 9; Congestion of Lungs, 1; Congestion of Brain, 4; Cold, 1; Consumption, 43; Convulsions, 46; Croup, 8; Delirium Tremens, 2; Decline, 4; Diarrhœa, 21; Diphtheria, 12; Disease of Bowels, 1; Disease of Brain, 3; Disease of Heart, 6; Disease of Liver, 4; Disease of Lungs, 6; Disease of Spine, 1; Disease of Throat, 2; Disease of Hip, 2; Dropsy, 6; Drowned, 4; Dysentery, 10; Erysipelas, 1; Fevers—childbed, 2; remittent, 17; scarlet, 8; typhoid, 38; typhus, 1; not stated, 12; Hemorrhage, 1; Hydrocephalus, 1; Inflammation, 2; Inflammation of Brain, 12; Inflammation of Bowels, 7; Inflammation of Kidneys, 5; Inflammation of Lungs, 3; Intemperance, 1; Marasmus, 2; Measles, 6; Neuralgia, 1; Old Age, 13; Poisoning, 2; Pleurisy, 2; Phthisis Pulmonalis, 1; Rheumatism, 1; Scrofula, 2; Still-born, 9; Summer Complaint, 38; Suicide, 1; Teething, 18; Tuberculosis, 2; Whooping Cough, 19; Worms, 1; Wound, 1; Uremia, 1; unknown, 37. Total, 1170.

*Nativities.*—Chicago, 331; other parts of the United States, 260; Bohemia, 24; Belgium, 2; Canada, 13; Denmark, 3; England, 39; France, 3; Germany, 223; Holland, 2; Ireland, 168; Norway, 44; Nova Scotia, 1; on the sea, 1; Poland, 1; Spain, 1; Sweden, 23; Scotland, 8; Switzerland, 1; Wales, 2; unknown, 20. Total, 1170.

*Ages.*—Under 5 years, 329; from 5 to 10 years, 95; from 10 to 20 years, 66; from 20 to 30 years, 202; from 30 to 40 years, 190; from 40 to 50 years, 116; from 50 to 60 years, 72; from 60 to 70 years, 47; from 70 to 80 years, 22; from 80 to 90 years, 5; from 90 to 100 years, 1; unknown age, 25. Total, 1170.

The deaths were divided throughout the city as follows: North Division, 311; South Division, 351; West Division, 503; unknown, 5. Total, 1170.

The number of deaths during the corresponding month of last year was 360.

